# Epidemiologic Characteristics of Domestic Patients with Hemorrhagic Fever with Renal Syndrome in Taiwan: A 19-Year Retrospective Study

**DOI:** 10.3390/ijerph17155291

**Published:** 2020-07-22

**Authors:** Chi-Jeng Hsieh, Chuan-Wang Li, Chun-An Cheng, Ding-Chung Wu, Wen-Chih Wu, Fu-Huang Lin, Yu-Ching Chou, Chia-Peng Yu

**Affiliations:** 1 Department of Health Care Administration, Oriental Institute of Technology, New Taipei City 220, Taiwan; fl004@mail.oit.edu.tw; 2 Department and Graduate Institute of Microbiology and Immunology, National Defense Medical Center, Taipei 114, Taiwan; 894020380@mail.ndmctsgh.edu.tw; 3 Institute of Preventive Medicine, National Defense Medical Center, New Taipei City 237, Taiwan; 4 Department of Neurology, Tri-Service General Hospital, National Defense Medical Center, Taipei 114, Taiwan; cca@ndmctsgh.edu.tw; 5 Department of Medical Records, Tri-Service General Hospital, National Defense Medical Center, Taipei 114, Taiwan; wuper@mail.ndmctsgh.edu.tw; 6 School of Public Health, National Defense Medical Center, Taipei 114, Taiwan; doctor0317@yahoo.com.tw (W.-C.W.); noldling@ms10.hinet.net (F.-H.L.); 7 Department of Surgery, Suao and Yuanshan Branches of Taipei Veterans General Hospital, Taipei 270, Taiwan

**Keywords:** hemorrhagic fever with renal syndrome (HFRS), hantavirus, longitudinal, surveillance

## Abstract

Background: Hemorrhagic fever with renal syndrome (HFRS) is an illness caused by hantaviruses. Numerous factors modify the risk of hantavirus transmission. This study explored the epidemiological characteristics, differences, and trends in terms of gender, age, season, and living areas of those diagnosed with domestically acquired HFRS in Taiwan from 2001 to 2019. Methods: We examined publicly available annual summary data on the domestic cases with HFRS from 2001 to 2019; these data were obtained from the web database of Taiwan’s Centers for Disease Control (CDC). Results: This study analyzed 21 domestic cases with HFRS from Taiwan’s CDC databases. In this study of the cases of HFRS in Taiwan, a gradual increase in the cases of those aged ≥40 years acquiring the disease was noted, and a distinct pattern of seasonal variation (spring) was observed. Furthermore, more men had domestically acquired HFRS, and living in Taipei metropolitan area (6 cases [28.6%]) and the rural areas (Gao-Ping region, 9 cases [42.9%]) was identified as a potential risk factor. This study represents the first report of confirmed cases of domestically acquired HFRS from surveillance data from Taiwan’s CDC, 2001–2019. Conclusion: This study highlights the importance of longitudinal studies covering a wide geographical area, particularly for highly fluctuating pathogens, to understanding the implications of the transmission of zoonotic diseases in human populations. Important data were identified to inform future surveillance and research efforts in Taiwan.

## 1. Introduction

Due to factors such as frequent international exchanges as well as a global climate and living environment changes, different emerging and re-emerging diseases are spreading rapidly. Incidence rates are increasing, particularly for vector-borne diseases. Hantavirus syndrome is a disease caused by infection with hantaviruses and is a type of zoonotic disease [1]. Hantaviruses, members of the order *Bunyavirales* of the *Hantaviridae* family, are round in shape; each virus has a diameter of approximately 100 nm and consists of three negative-sense single-stranded ribonucleic acid (RNA) segments [2]. The viruses have a lipid membrane and are easily deactivated by oil-dissolving solvents such as alcohol, general disinfectants, and household bleach. Through gene sequence alignment and serodiagnosis, hantaviruses are currently categorized into more than twenty types; some have different geographical distributions and are unique to different rodent hosts. Some hantaviruses are not infectious to humans, whereas others are associated with human diseases. Such human viruses are mainly classified into two categories, one being the main cause of hantavirus hemorrhagic fever with renal syndrome (HFRS), mostly found in Asia and Europe, and the other being the cause of hantavirus pulmonary syndrome (HPS) [3]. The five types of viruses responsible for HFRS are as follows: (1) Hantaan viruses, carried by *Apodemus agrarius* (field mice); (2) Seoul viruses, carried by *Rattus norvegicus* and *R. rattus*, (rats); (3) Puumala viruses, carried by *Myodes glareolus* (bank voles); (4) Dobrava viruses, carried by *Apodemus flavicollis*, (yellow-necked field mice) [4]; and (5) Saaremaa viruses, carried by *Apodemus agrarius* [5]. Of these viruses, symptoms caused by Hantaan and Dobrava viruses are the most severe, with fatality rates between 5% and 10% [4], followed by Seoul viruses (fatality rate: 1% to 2%). Patients infected with such viruses mostly have hemorrhage and renal dysfunction, which distinguishes them from the other four types. Furthermore, Seoul virus infections are more likely to occur in cities due to the behavioral habits of their hosts. The symptoms generated by Puumala viruses and Saaremaa viruses are the mildest, with the fatality rate being less than 1% [6].

HFRS first emerged during World War I. The first case was reported in Soviet Russia in 1913. Cases were also reported in Japan and Sweden from 1932 to 1934, with further cases confirmed from 1951 to 1954 during the Korean War. According to the World Health Organization, 60,000 to 100,000 HFRS cases are reported every year, and HFRS cases occur mostly in Asia and Europe. The prevalent serotypes in Asia are Hantaan viruses and Seoul viruses, and those in Europe are Puumala viruses, Dobrava viruses, and Saaremaa viruses. Most cases in Asia are reported in China and Korea. Concerning cases in China [7], HFRS is prevalent in Central and Southern China, with a total of 100,000 to 200,000 cases reported in the early 20th century. In recent years, China has taken relevant precautions, and confirmed cases have accordingly decreased. Since 2007, the total number of cases reported every year has been approximately 11,000 to 13,000; the majority (82.5%) of these cases have been concentrated in North and Northeast China. The People’s Republic of China instituted from 1950 a unique official registry, first with medically defined HFRS cases, and then later (from the early 1980s) medically and serologically confirmed HFRS cases. Through 2014, this yielded an unsurpassed total of 1,625,002 HFRS cases, and 46,968 (2.89%) fatalities [8]. Cases in Europe are prominently reported in Western and Northern Europe. Studies have indicated that the total number of cases reported in Europe is approximately 3000 (this differs according to country and year). Regarding cases in Germany [9], substantial HFRS outbreaks occurred in 2007, 2010, and 2012, with those in 2012 being the most severe. More than 2800 HFRS cases were reported, which were mainly associated with Puumala virus serotypes. The primary cause only suggested that global warming may lead to an increase in infections in the future. 

Rodents are a natural host of hantaviruses, and each hantavirus has a specific rodent host. Humans are incidental hosts [10]. The host of Hantaan viruses is *A. agrarius*, which can be found in Asia, and the host of Seoul viruses is *R. norvegicus*, which is distributed worldwide, being found in fields or areas of human habitation. HFRS is mainly transmitted through the inhalation of rodent secretion, excreta, and droplets into the respiratory tract [11]. The virus exists in the urine, droppings, and saliva of infected yet asymptomatic rodent hosts, and a high concentration of viruses can also be found in their lungs. People may be infected after inhaling air or making contact with objects contaminated with the viruses or from being bitten by infected rodent hosts. International studies have demonstrated that those of certain occupations, such as pet rat owners, have higher risks of hantavirus infection [12]. Among the various viruses that cause HFRS, transmission from one human to another has not yet been identified. 

The incubation period of HFRS is approximately 12 to 16 days; however, this can vary from 5 to 42 days. The typical initial symptoms are a sudden fever lasting 3 to 8 days, and this may by followed by conjunctival hyperemia, weakness, backache, headache, abdominal pain, anorexia, vomiting, and facial flushing [13]. Hemorrhagic symptoms appear on the third to sixth day; later symptoms may include proteinuria, hypotension, shock, and mild kidney diseases. Over the course of the disease, acute kidney failure may arise and lasts several weeks, with a corresponding fatality rate of 5% to 15%. The course of HFRS is divided into five stages: febrile, hypotensive, oliguric, polyuric, and convalescent. Stages may occasionally overlap. 

Taiwan is an island nation; therefore, infectious diseases are mostly transmitted through seaports and airports, with rodent-borne viruses being one transmission source. In 1995, Taiwan discovered its first HFRS case that originated from China. Cases since 2001 have been regarded as sporadic, with massive outbreaks rarely seen. Additionally, Taiwan’s Centers for Disease Control (CDC) established the Taiwan National Infectious Disease Statistics System (TNIDSS) in 2001 [14], providing the public, academics, and those in the media with access to the latest domestic epidemic updates related to HFRS. Accordingly, this study used the system to investigate the epidemiological characteristics, differences, and trends in terms of sex, age, season, and living areas related to domestic HFRS cases in Taiwan from 2001 to 2019.

## 2. Materials and Methods

### 2.1. Ethical Policy

Studies using information that is freely available in the public domain and that analyze open data sets where data have been properly anonymized do not require ethical approval. The authors are sure about the added value of this study that conforms with the public use of the government reports.

### 2.2. Definition of Confirmed Cases

The central competent authority in Taiwan classified HFRS as a Category 3 communicable disease, as listed in the Communicable Control Act in 2001. It was reclassified as a Category 2 disease in 2004. The following procedure should be followed for communicable diseases listed in this Act’s documentation. Whenever infection with a disease occurs in an individual, doctors or medical institutions should report this to health authorities within legal time limits. Appropriate treatment should be administered, and isolation measures can be applied in accordance with the Act’s stipulations. The listed communicable diseases usually have a rapid transmission speed as well as high hazard severity and fatality rates. Therefore, when the following conditions are observed or suspected during inspection, doctors should report information to relevant health authorities within 24 h, as stipulated in the regulations below: Acute fever along with varying severity levels of hemorrhagic diseases and abnormal renal function, and having two or more of the following symptoms: headache, muscle soreness or backache, nausea or vomiting, diarrhea, and blurred vision; having a relevant travel history; having contact with rodents or their excreta and secretion; having probable or confirmed contact with contaminants or a history of such contact; having conducted experiments on hantaviruses or relevant specimens. Furthermore, patients having undergone inspections in hospitals and received positive results from the following analyses: (1) molecular biology analysis of clinical specimens, (2) hantavirus-specific antibodies IgM in acute-phase serum, (3) antivirus-specific antibodies IgM or IgG (either one) having seroconversion or a 4-fold (or higher) increase, (4) disease confirmed by immunohistochemical analysis from a biopsy**.** Reports from Taiwan’s CDC have indicated that a confirmation of an HFRS case should be made in accordance with the receipt of positive results in any of inspections (1), (3), or (4).

### 2.3. Data Source

This study employed TNIDSS, a public database established by Taiwan’s CDC [14]. The public TNIDSS database includes data on five categories of communicable diseases listed in the Communicable Control Act literature. The website is updated with the latest epidemic information early each morning, providing the latest charts (e.g., run charts), reports (in Excel format), distributions (in county, city, or township), and encyclopedias of different disease types. This allows for epidemic information to become more transparent and up to date. To ensure information security and privacy, the public database includes only secondary data (i.e., notification date, onset date, confirmation date, and the number of confirmed domestic and imported cases imported of HFRS) without case details. The database does not contain the medical history of patients, their signs and symptoms, or the results of laboratory data.

### 2.4. Data Analysis

This is a retrospective historical study of all domestic HFRS cases since 2001. We confirmed the number of people diagnosed as having HFRS from 2001 to 2010 and from 2011 to September, 2019 and examined the distribution of their epidemiological characteristics (sex, age, time of diagnosis, living area), differences, and results. Next, we emphasized sex, age, time of diagnosis, living area change, trends, and related results in our analysis of cases of HFRS from 2001 to 2006, from 2007 to 2012, and from 2013 to 2019. Descriptive data are shown as mean and summary, where appropriate. Categorical variables were compared using the chi-square test. All statistical analyses were performed using SPSS (IBM SPSS version 21; Asia Analytics Taiwan Ltd., Taipei, Taiwan). All statistical tests were 2-sided with an α value of 0.05. *p* values of < 0.05 were considered to represent statistical significance.

## 3. Results

### 3.1. Study Population

During the study period, 21 adults (Table 1) had confirmed diseases, 18 (85.7%) of which were men. Among them, 20 (95.2%) patients were 20–64 years old and 1 (4.8%) was >65 years old. The patients (10 in spring, 5 in summer, and 6 in winter) were examined and screened for HFRS. The characteristics of patients with confirmed cases (including habitual residence) are shown in Table 2.

### 3.2. Epidemiological Features

**Figure 1:**
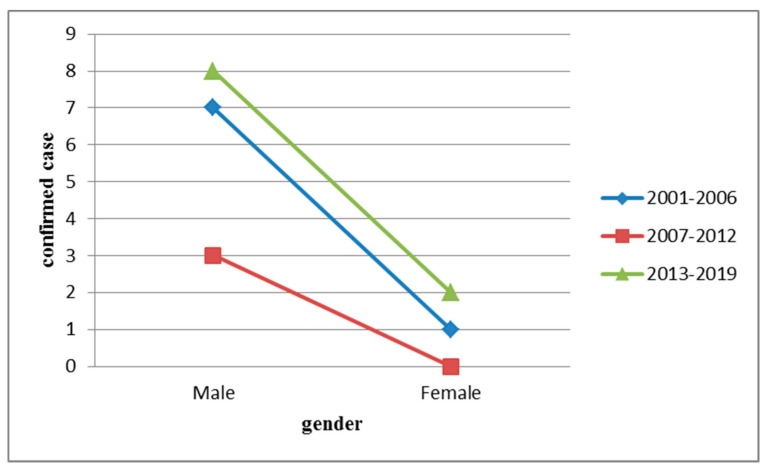
Gender distribution of hemorrhagic fever with renal syndrome (HFRS) in indigenous confirmed cases, Taiwan, 2001–2019.

HFRS was detected more often in men in 2001–2006 (33.3% [7/21]), 2007–2012 (14.3% [3/21]), and 2013–2019 (38.1% [8/21]) than in women (4.8% [1/21], 0% [0/21], and 9.5% [2/21], respectively; Figure 1).

**Figure 2:**
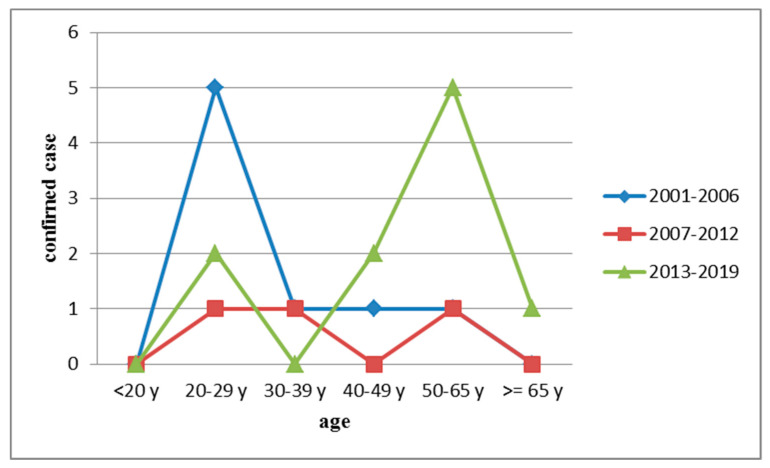
Age distribution of hemorrhagic fever with renal syndrome (HFRS) in indigenous confirmed cases, Taiwan, 2001–2019.

HFRS was confirmed more often in individuals in the age range of 20–29 years (23.8% [5/21]) and in those aged 50–65 years (23.8% [5/21]; Figure 2).

**Figure 3:**
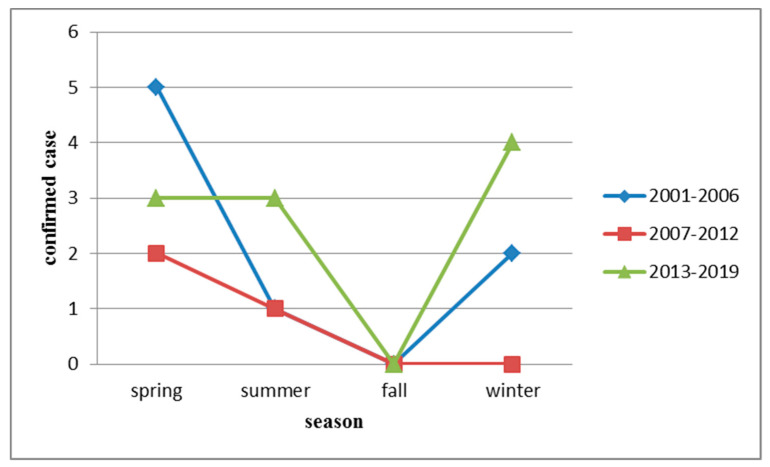
Seasonal distribution of hemorrhagic fever with renal syndrome (HFRS) in indigenous confirmed cases, Taiwan, 2001–2019.

HFRS infections were identified throughout the year except during fall, with the highest incidence in spring followed by winter (Figure 3).

**Figure 4:**
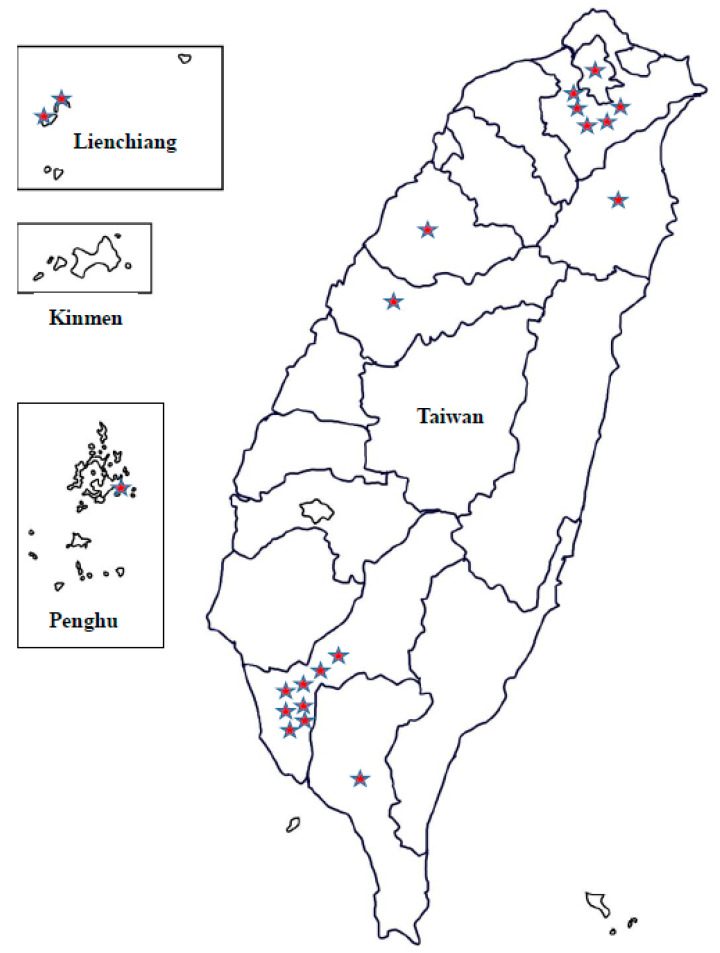
Geographic distribution of hemorrhagic fever with renal syndrome (HFRS) in indigenous confirmed cases, Taiwan, 2001–2019.

**Figure 5:**
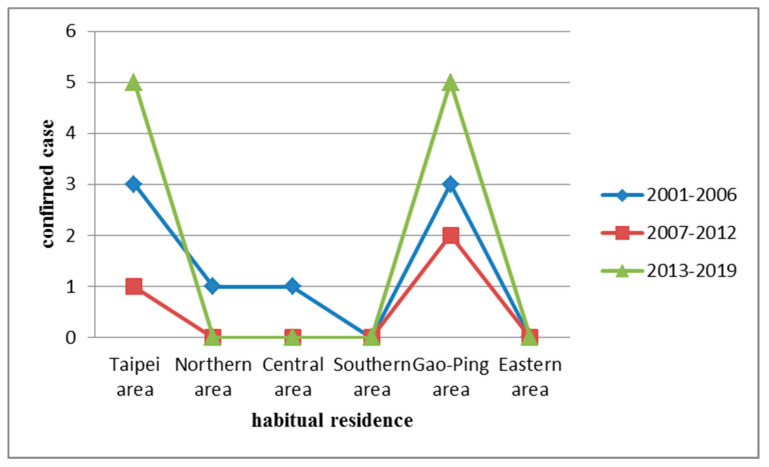
Habitual residence distribution of hemorrhagic fever with renal syndrome (HFRS) in indigenous confirmed cases, Taiwan, 2001–2019.

HFRS infections were identified in all residential regions in Taiwan (highest incidence in the Taipei and Gao-Ping areas; Figure 4 and Figure 5).

## 4. Discussion

Zoonoses are diseases that can spread from animals to humans and from humans to animals [15]. Pathogens can be transmitted directly between humans and animals or carried by vectors to enter another organism [16]. Zoonoses can be categorized into viruses, bacteria, molds, parasitic worms, and protozoa; they can be transmitted though contact, inhalation, or consuming foods and drinks that contain pathogens [17]. Transmission of zoonoses can be a threat to human health. HFRS is a zoonotic disease transmitted by rodent hosts [18]. From 2001 to the time of writing, 21 confirmed domestic cases of HFRS (Table 1) and 1 imported (from China) confirmed case (December, 2007) have been reported. In the past 10 years, 0–4 cases were reported yearly, which can be considered to be sporadic, especially in terms of the patients’ occupation; accordingly, the susceptible population cannot be determined. In general, the number of cases depends on rodent distribution in the surrounding area and the likelihood of contacting hantaviruses directly or indirectly. Based on the prevalence in Taiwan literature [19,20,21], various rodent species serve as potential hosts of hantaviruses, including *Rattus flavipectus*, *Rattus losea*, *Rattus norvegicus*, *Rattus tanezumi*, *Bandicota indica*, *Mus musculus*, and *Apodemus agrarius* of the order Rodentia, along with *Suncus murinus* of the order *Insectivora*. Of these eight species, *Suncus murinus*, *Rattus norvegicus*, *Mus musculus*, and *Rattus tanezumi* are easily found in environments (residences) where human activities take place. According to an investigation on rodent-borne diseases within the five metropolises in Taiwan, the dominant rodent species in markets and night markets are *Suncus murinus* (52.9%) and *Rattus norvegicus* (45.4%). The larger the rodents are, the more likely their HFRS antibodies being tested positive becomes. *Rattus norvegicus* rodents possess the highest HFRS antibody sensitivity (20.1%); those captured in Taipei City had the highest sensitivity (23.9%). Seoul serotypes (100%) are the main HFRS antibodies. Through molecular biology analysis, it was revealed that the hantavirus carrier rates of *Rattus norvegicus* and *Suncus murinus* were 80.0% and 40.0%, respectively [21]. *Suncus murinus* (the house shrew) as a hantavirus carrier, the soricid-borne hantavirus Thottapalayam virus (TPMV), has not been proven so far to be pathogenic for humans [22]. This study suggested that the Seoul virus is the main pathogenic hantavirus prevalent in Taiwan. No statistical differences were found between rodent species and their geographical distribution. However, people unfamiliar with rodent-borne diseases are less willing to participate in deratting, which increases difficulty of prevention work. The public health interventions are currently that the government promotes autonomous environmental management and biological control in Taiwan (for example: *Milvus migrans*). Large-scale rat prevention measures can function as urgent precautions against outbreaks [23]. Market management teams should include waste removal, setting up rat bait and traps, and clearing out garbage shortly after business hours as stallholder contract stipulations; vendors should seal food and gutters should be covered with lids of appropriate apertures to lower rodent density and reduce the likelihood of human–rodent contact. Public health education should also be emphasized to help people understand the importance of rat prevention, learn more about rodent-related diseases, and maintain high sanitation levels in their surroundings. The public should ensure no rat infestation, no rat dwelling, and no rat feeding to fundamentally prevent rodent density from increasing and thus ensure public health and safety [24].

HFRS is a highly infectious viral disease. Humans can be infected by inhaling droplets containing rodents’ urine, saliva, and droppings. Fleas, ticks, and mosquitos do not transmit hantaviruses. Domestic HFRS cases are reported in Taiwan. With the increase of international travel, import-related threats remain, particularly from neighboring regions where HFRS is prevalent, including China and Southeast Asian countries. Since 2001, only 1 imported case has been reported [14]. To prevent imported infectious diseases from spreading and affecting people’s health, epidemic prevention inspectors stand guard at all international seaports and airports on public holidays and remind outbound travelers to cooperate with fever inspection personnel when returning. Furthermore, travelers with fever, cough, vomiting, diarrhea, rash, and jaundice should report to inspectors. Foreign travelers who have symptoms within 15 days after returning home should seek medical advice immediately; travel and contact history should be provided for diagnosis references so that diagnosis can be confirmed as soon as possible, treatments can be conducted in a timely manner, and epidemics can be avoided. 

A previous study indicated that HFRS infections are most often diagnosed in those of the male sex. It is suggested that this is due to increased exposure as a result of men’s occupational and recreational activities [25]. According to data on domestic HFRS cases over the years, more men work as butchers, fisherpersons, fishmongers, and in the industries of stock farming, aquaculture, and agriculture. Among these occupations, recognition and notification of symptoms are well established. Fewer cases of female infections have been identified, which may be due to more women working in indoor environments; similar results have been identified in international studies [26,27]. No statistically significant differences were noted in terms of sex, which accords with the findings of international foreign studies [28,29]. Cases of male exposure outnumbered those of female exposure from 2013 to 2019. During this time, the male incidence rate was 80% (8/10), which also corresponds with the results of international studies [30,31]. 

A related study confirmed a high lifetime risk for both women and men for acquiring HFRS infections in endemic areas [30]. The present study demonstrated that no significant differences were observed in a comparison of HFRS cases over the years in terms of age, only that number of HFRS cases in patients aged 40 to 49 years increased over the past 6 years. Furthermore, HFRS cases in patients aged 50 to 65 years reached a peak during 2013–2019, indicating results similar to those of international studies [30,32,33,34]. 

Climate could also influence the population abundance of host rodent and hantavirus transmission dynamics. Temperature might also affect reproduction and survival rates of small rodents as well as the length of time that the virus remains infectious in the environment [35]. No statistical differences were observed when domestic HFRS cases were compared in terms of seasons. More cases were reported in spring and winter than in other seasons, and the largest number of cases identified was during 2001–2006 and 2013–2019, which corresponds with results of related international studies [32,33]. 

Furthermore, in accordance with previous findings, a strong relationship was observed between living in rural, stock farming areas or close to farms and being at risk of hantavirus infection [36,37]. Risk factors such as living in close contact with rodents, agriculture, and stock farming are more likely in rural areas [37,38]. Although most HFRS cases have been reported in rural settings and in Europe or Asia, there are important exceptions. In fact, the Seoul serotype has been reported mainly during urban, rather than rural outbreaks [39]. No significant differences were observed in a comparison of domestic HFRS cases in terms of living area, although more cases were reported in the Taipei metropolitan area and the rural Gao-Ping area than in other regions; furthermore, the cases reported during 2013 to 2019 account for 48% of all HFRS cases, the highest proportion over the years of monitoring. This is similar to the result in international literature: metropolitan areas and rural agricultural or stock farming areas have a higher risk of having HFRS outbreaks [30,34,40,41]. Because these counties are distributed throughout Taiwan, we can assume that infected individuals are found throughout the island nation, which suggests that hantavirus diseases could be an emerging health problem.

Evidence is required to determine the next actions and decisions to ensure the execution of appropriate public health policy [42]. For example, in this study, researchers observed and recorded HFRS relevant data on the TNIDSS public database and selected credible information from large medical databases by employing epidemiological and statistical methods. The researchers applied all acquired medical evidence on public health services. As a result, it is expected that the outbreaks of acute infectious diseases (e.g., HFRS) will be minimized or eliminated. Through the enactment of preventive healthcare standards based on public health science, high-quality health risk assessments were provided to reduce unnecessary medical resource expenditure. Furthermore, the core value of public health policy is disease prevention. According to the natural history of a disease, the preventive health care strategy is divided into three levels (primary, secondary, and tertiary prevention) and five stages. The researchers of this study conducted primary prevention, explored epidemiological characteristics and trends, assessed exposure risk, and attempted to modify the lifestyles of the susceptible population to enhance public health.

The concept of “one health” relates to the relationship between the health of humans and animals [43]. In terms of HFRS, if rodents carry pathogens or are infected with hantavirus diseases, humans may also be infected. Furthermore, with environment changes and deterioration caused by human activities, the growth and decline of species, or their health conditions, may also affect human society. Each organism has a role in the interconnected Earth; once a link is broken, it affects the ecosystem and human survival. These visionary public health concepts can become references for governments and public health experts worldwide for enacting health policies or distributing health care resources.

Three limitations of this study should be noted. First, the Taiwan’s CDC data only provide the basic epidemic data of patients with HFRS and not any clinical data, and did not provide any detailed experimental procedures. However, this study is convinced of the authenticity of the positive confirmed cases announced by Taiwan’s CDC. However, the researchers could not compare clinical data to determine differences or trends related to HFRS symptoms. Second, public databases do not offer the genotypes of hantavirus virus strains; therefore, the virus strain that is currently prevalent or phylogenetic differences between domestic and imported virus strains could not be determined. Third, public health policy changes are not being proposed based on the results from this study. However, this study is unique, owing to the abundant data the government network platform provides (even in the early construction stage). Public databases have been properly maintained over the years; this facilitates research and increases the value of the data. Related institutions also conduct monitoring related to disease types or symptoms and lead to further follow-up studies.

## 5. Conclusions

This is the first report in Taiwan concerning the epidemiological characteristics and trends of HFRS cases from 2001 to 2019. In this study on the incidence of HFRS in Taiwan, a gradual increase in incidence was noted with patient age and a distinct pattern of seasonal variation was determined. Furthermore, more men were diagnosed as having domestically acquired HFRS than women, and living in the Taipei metropolitan area or rural areas was suggested as a potential risk factor. Furthermore, it is highly recommended to extend HFRS vaccination to people older than 50 years to better protect them against the disease. This information will be of value to policymakers and clinical experts for direct prevention and control activities related to hantaviruses that cause the most severe illnesses and have the greatest burden on the Taiwanese health care system. This study highlights the importance of longitudinal studies encompassing wide geographical areas, particularly for highly fluctuating pathogens and their reservoirs, to understanding the implications of the transmission of zoonotic diseases in human populations. Crucial data were identified to inform future surveillance and research efforts in Taiwan.

## Figures and Tables

**Table 1:** Descriptive statistics of hemorrhagic fever with renal syndrome (HFRS) in indigenous confirmed cases, Taiwan, 2001–2019.

Years	Summary Cases (%)
2001	2 (9.5)
2002	Not applicable
2003	Not applicable
2004	3 (14.3)
2005	Not applicable
2006	3 (14.3)
2007	Not applicable
2008	1 (4.8)
2009	Not applicable
2010	1 (4.8)
2011	Not applicable
2012	1 (4.8)
2013	Not applicable
2014	2 (9.5)
2015	2 (9.5)
2016	4 (19.0)
2017	Not applicable
2018	1 (4.8)
2019	1 (4.8)

**Table 2:** Epidemiological features of hemorrhagic fever with renal syndrome (HFRS) in indigenous confirmed cases, Taiwan, 2001–2019.

Variables	HFRS Cases (*n* = 21)		*p* Value
	2001–2010	2011–2019	
Gender			1.000
Male	9	9	
Female	1	2	
Age			
<20	0	0	Not applicable
20–64	10	10	1.000
>=65	0	1	1.000
Season			
Spring	7	3	0.086
Summer	1	4	0.311
Fall	0	0	Not applicable
Winter	2	4	0.635
Area			
Northern	5	5	1.000
Central	1	0	0.476
Southern	4	6	0.670
Eastern	0	0	Not applicable
